# Quantifying Differences in Heritability among Psoriatic Arthritis (PsA), Cutaneous Psoriasis (PsC) and Psoriasis vulgaris (PsV)

**DOI:** 10.1038/s41598-020-61981-5

**Published:** 2020-03-18

**Authors:** Quan Li, Vinod Chandran, Lam Tsoi, Darren O’Rielly, Rajan P. Nair, Dafna Gladman, James T. Elder, Proton Rahman

**Affiliations:** 1 Department of Medicine, Faculty of Medicine, Memorial University, St. John’s, Newfoundland, St. John’s, Newfoundland and Labrador A1B 3X9 Canada; 2 Department of Medicine, Division of Rheumatology, University of Toronto, Toronto, Ontario M5S 1A8 Canada; 3 Department of Laboratory Medicine and Pathobiology, University of Toronto, Toronto, Ontario M5S 1A8 Canada; 4 Institute of Medical Science, University of Toronto, Toronto, Ontario M5S 1A8 Canada; 5 Krembil Research Institute, University Health Network, Toronto, Ontario M5S 1A8 Canada; 6 Department of Dermatology, University of Michigan Medical School, Ann Arbor, Michigan 48109 USA; 7 Department of Computational Medicine & Bioinformatics, University of Michigan, Michigan, USA; 8 Department of Biostatistics, University of Michigan, Michigan, USA; 9 Ann Arbor Veterans Affairs Hospital, Ann Arbor, MI USA

**Keywords:** Psoriatic arthritis, Heritable quantitative trait, Skin diseases

## Abstract

Chronic plaque psoriasis and psoriatic arthritis are multifactorial inter-related diseases with strong genetic contributions. Better elucidation of the heritability of psoriatic disease subsets is important for identifying novel genes, risk stratification and potential clinical applications. In this study, we used two mixed-effect modelling methodologies to assess the additive contribution of common single nucleotide polymorphisms from genome-wide association studies to estimate the heritability of cutaneous psoriasis, psoriasis vulgaris and psoriatic arthritis. We found that cutaneous psoriasis and psoriatic arthritis both exhibit considerable heritability, with a greater contribution coming from cutaneous psoriasis.

## Introduction

Chronic plaque psoriasis (henceforth termed ‘psoriasis’) is a complex immune-mediated cutaneous disease. Psoriatic arthritis (PsA) is an inflammatory arthritis that occurs in approximately 20 to 30% of patients with psoriasis^1,2^. Although the etiology of psoriatic disease is not fully elucidated, it is widely viewed as a multifactorial disease as there is substantive evidence to implicate an interaction between inherited genetic factors and environmental triggers^2^. PsA can be thought of as a distinctive entity within a broader disease (i.e., psoriasis). To investigate the genetics of psoriatic disease, it is prudent to phenotypically segregate this entity into three subsets: psoriasis in the absence of arthritis (cutaneous psoriasis, PsC), inflammatory arthritis with psoriasis (psoriatic arthritis, PsA) and psoriasis irrespective of inflammatory arthritis (psoriasis vulgaris, PsV). Under this classification, PsA or PsC alone represents a more homogenous group than PsV. Defining such homogeneous phenotypes will facilitate the elucidation of disease-related genes and relevant signalling pathways specific to cutaneous and articular manifestations of psoriatic disease.

Heritability essentially refers to how much variation in a trait is due to variation in genetic factors. Phenotypes vary within a population due to environmental exposure and genotypes at particular loci. The combined effect of all loci is the genotypic burden or value due to the additive effect of all loci, and possible allelic interactions within a locus (dominance) and between loci (epistasis)^3^. Broad-sense heritability (H^2^) refers to the proportion of phenotypic variation due to genetic value including effects due to dominance and epistasis. In contrast, narrow-sense heritability (h^2^) is the proportion of genetic variation due to additive genetic values, and represents the heritability measure most commonly used in human complex disease studies^3^.

Population-based genetic epidemiological studies, immunogenetics, candidate genes and more recently GWAS studies have demonstrated a strong genetic basis for psoriatic disease as previously reviewed^4^. Population-based genetic epidemiological studies quantitate familial aggregation using prevalence of affected first degree relatives of probands compared with prevalence of affected first degree relatives of unaffected controls^5^. The recurrence rate of PsA among first degree relatives of PsA probands (λ_s_ 30 to 48) is greater than the recurrence of psoriasis among first degree relatives of PsV probands (λ_s_ 4 to 10)^4,6–9^. Reliance on the population prevalence of disease is a major limitation of familial aggregation estimates based on population risk. Underestimating the prevalence of PsA in the general population can inflate the familial aggregation estimate when calculating the sibling recurrence risk score (λ_s_). This is of particular relevance in PsA as estimates for population prevalence in the literature vary widely from 0.1% to 0.5%^2,10,11^. Notably, the prevalence in siblings of probands with PsA compared to siblings of probands with PsC was similar in these studies, indicating that the genetic contribution is driven mainly by psoriasis. Genetic contribution to complex disease can also be estimated from twin studies by comparing concordance of monozygotic and dizygotic twins^12,13^. Although there are no large PsA twin studies reported in the literature, for PsV the monozygotic twin concordance rate is almost three times higher (62 to 70%) compared with dizygotic twins (21 to 23%) resulting in heritability estimates (h^2^) between 60% and 90%^14^.

Over 60 genetic signals have reached a genome-wide level of significance from genome-wide association scan (GWAS) studies in psoriasis, whereas ~20 genetic signals have achieved the same in PsA cohorts as previously reviewed^15^. The lack of PsA-specific genes identified from GWAS studies combined with the considerable clinical heterogeneity of PsA and the lack of cases in PsA GWAS studies (compared with psoriasis), suggest that additional GWAS on larger PsA cohorts followed by meta-analyses should identify additional PsA-specific variants or that the genetic burden (or variance) for PsA is just not as high as originally thought. Recently, algorithms have been developed to assess heritability of complex disease through unbiased estimates of the variance explained from genome-wide arrays or whole-genome sequencing. The three most widely used single nucleotide polymorphism (SNP)-based methods to estimate heritability are genomic relatedness matrix residual maximum likelihood analysis (GREML) in the genome-wide complex trait analysis package (GCTA)^16^, linkage disequilibrium (LD) adjusted kinships approach (LDAK)^17^, and LD score regression^18^. In this study, we set out to assess the heritability of PsC, PsA and PsV by interrogating SNPs from large-scale genotyping arrays. Better approximation of the heritability of PsC, PsV and PsA will culminate in more efficient genetic profiling of psoriatic disease and facilitate gene identification studies by providing more accurate estimates of sample sizes needed based on the heritability of different subsets of psoriatic disease.

## Methods

The data used for this study were obtained from a previously published GWAS study in psoriatic disease^19,20^. Table 1 lists the characteristics of patients and controls in this study. All psoriatic disease samples and controls were of European Caucasian descent. The PsV group included any subject diagnosed with psoriasis by a dermatologist, irrespective of the present of inflammatory arthritis. The PsC group included only subjects with cutaneous psoriasis for at least 10 years in the absence of any previous or current inflammatory arthritis. The PsA group included psoriasis subjects diagnosed with inflammatory arthritis diagnosed by rheumatologists. All PsA subjects satisfied the CASPAR criteria^21^. The heritability of PsC, PsV and PsA were estimated by interrogating 2938 PsV, 1155 PsC, 715 PsA subjects and 3117 unaffected controls.

**Table 1:** Demographics for all psoriatic patients and controls in the study.

Characteristic	PsA	PsC	PsV	Controls
Number	715	1155	2938	3117
Age	52.49 (±13.4)	51.09 (±15.1)	49.87 (±16.4)	42.64 (±17.6)
Female	53%	51%	52%	58%
Male	47%	49%	48%	42%
Psoriasis_age_at_onset	28.34 (±15.5)	22.76 (±13.8)	28.56 (±17.2)	—
Psoriasis_duration	24.19 (±15.3)	28.33 (±13.7)	21.31 (±15.8)	—
PsA_age_at_onset	38.44 (±13.9)	—	—	—
PsA_duration	14.49 (±12.3)	—	—	—

All samples were genotyped on a custom Axiom Biobank plus genotyping array with 461,092 autosomal SNPs. Stringent quality control (QC) was performed and genetic markers with a high missing rate (>1%), low minor allele frequency (MAF < 5%), or significant deviation from Hardy-Weinberg equilibrium (p < 0.001) were filtered, with 230k SNPs remaining for heritability estimation. SNPs were imputed based on

autosomal reference panel of HapMap Phase 3 (HM3) CEU cohort with 1.5 million sites. After setting imputation quality cut-off as >0.9, 735k high quality SNPs remained. SNPs with low MAF (<5%) and SNPs with significant deviation from Hardy-Weinberg equilibrium (p < 0.001) were excluded, leaving a total of 401k imputed SNPs for the heritability estimation. Since the major histocompatibility complex (MHC) region has a large impact on the SNP heritability estimation for psoriatic disease and related phenotypes, heritability was also estimated after removing SNPs located within the MHC region (chr6:25–35 Mb).

In this study, the heritability was estimated using the typed SNPs. This SNP heritability (h^2^_SNP_) is defined as the proportion of total phenotypic variation due to additive genetic effects of a given set of typed SNPs, and can be calculated as ratio of the variance of total genetic effects to the total phenotypic variance. The effects of non-additive genetic variation and environment will not confound the h^2^_SNP_. These variance components were estimated using genotypic relatedness matrix (GRM) and the restricted maximum likelihood (REML) approach. The heritability of PsC, PsV and PsA in our analysis was estimated using the LDAK and GCTA methods. The LDAK and GCTA methods both rely on the restricted maximum likelihood algorithm (ReML). However, these two approaches have different assumptions for the relationships between the expected SNP heritability and allele frequency, levels of LD and genotype certainty.

In LDAK, the SNP effects are a function of LD and weakly inversely proportional to the minor allele frequency (MAF): the SNPs were re-weighted to account for LD; SNPs in low LD contribute more than those in high-LD regions; higher-quality SNPs contribute more than lower-quality ones, while in the GCTA method: these effects are independent of LD and strongly inversely proportionate to MAF, also GCTA’s heritability does not vary with genotype certainty. These two methods both have their limitations and are sensitive to assumptions about genetic architecture^22^. In our analysis, both approaches are presented, and both lead to the same overall conclusions.

The estimation of heritability requires the samples with similar ancestry. The potential population stratification will affect the estimation. We performed principal components analysis(PCA) on our samples with genotype data^23^. In the heritability estimation, to account the population stratification, we included first five eigenvectors from PCA as covariates. We also included sex as a covariate to control the sex effect. Parallel analyses were performed after removing SNPs from the MHC region. For case–control studies, the heritability estimation can be biased by the prevalence of the disease in the population. Thus, the population prevalence was varied from 0.5% to 4.0% (0.5%, 1%, 2%, 3% and 4%) to assess the robustness of the heritability estimations.

## Results

**Figure 1:**
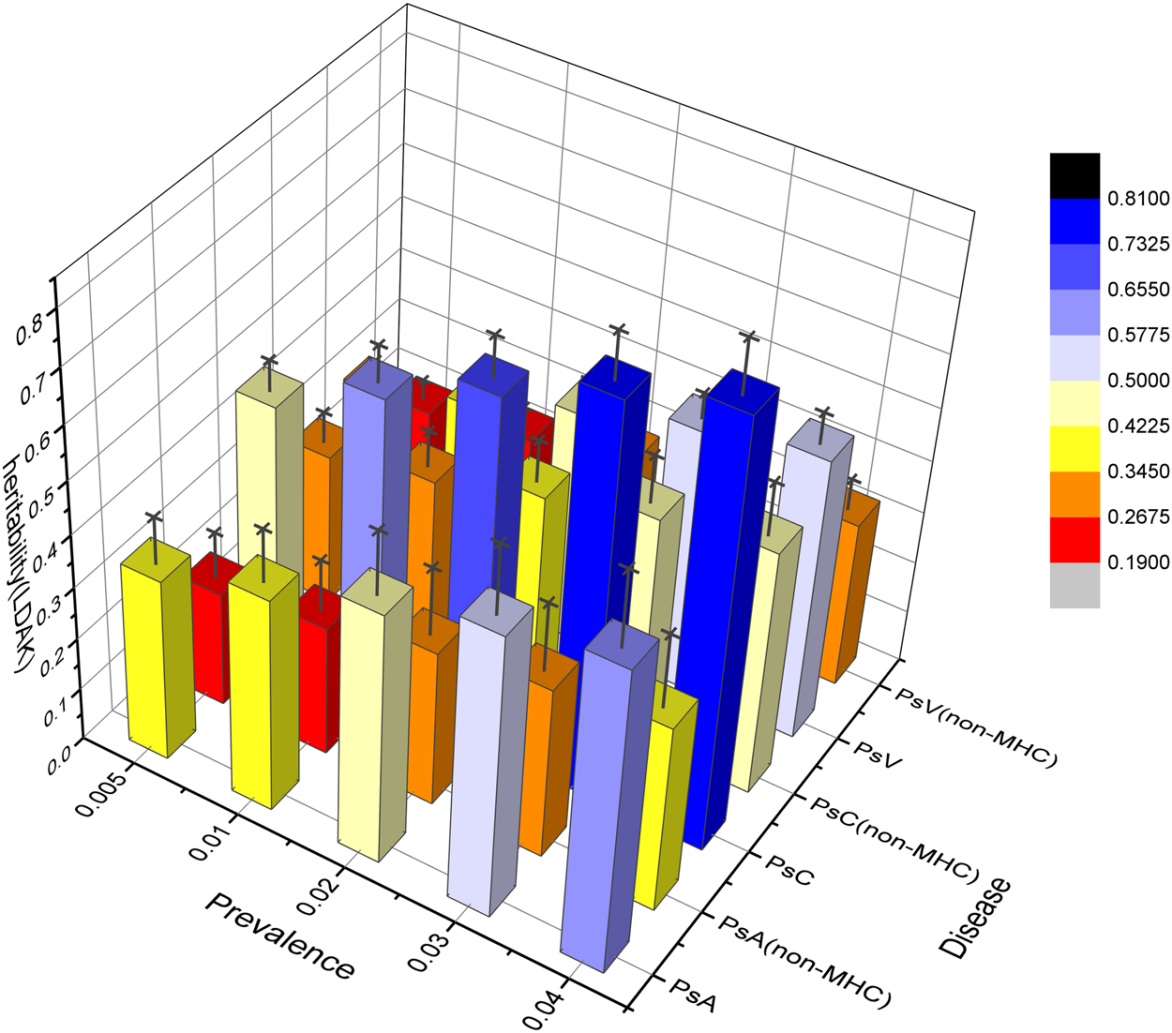
3D bar plot of heritability estimation including and excluding MHC SNPs using the LDAK method adjusted by prevalence. The SNPs are genotyped. The X-axis is disease prevalence from 0.5% to 4%, Y-axis is diseases and SNP regions, Z- axis is heritability score, standard deviation is showed as black error bar.

The heritability assessment for psoriatic disease using both the LDAK and the GCTA methods are presented with and without imputation and including and excluding SNPs located within the MHC region. Although the heritability estimates vary depending on the LDAK and the GCTA methods, there are numerous similarities. Using the LDAK method with 230k SNPs from GWAS data and adjusted with a prevalence as 1%, the heritability of PsC (h^2^ = 0.58) was greater than both PsV (h^2^ = 0.37) and PsA (h^2^ = 0.41) (Table 2). A similar trend was noted with SNP imputation (Table 2). Using the LDAK method and 401k imputed SNPs, the heritability of PsC (h^2^ = 0.50) was greater than both PsV (h^2^ = 0.32) and PsA (h^2^ = 0.43) (Table 2). Excluding SNPs located within the MHC region and using the LDAK method, the heritability of PsC (h^2^ = 0.29 vs 0.33) was greater than both PsV (h^2^ = 0.19 vs 0.22) and PsA (h^2^ = 0.25 vs 0.28) with and without SNP imputation (Table 2**;** Fig. 1). This trend is maintained as disease prevalence is varied from 0.5 to 4.0% (Fig. 1). As expected, use of all SNPs resulted in a greater heritability score compared with non-MHC SNPs with a considerable proportion of the entire heritability (h^2^) being contributed from the SNPs located within the MHC region.

**Table 2:** Heritability estimation for psoriatic disease (adjusted with prevalence 1%) using both the LDAK and the GCTA methods presented with and without imputation and including and excluding SNPs located within the MHC region.

h^2^ method	SNPs	Genotyped			Imputed		
		PsA	PsC	PsV	PsA	PsC	PsV
LDAK	All SNPs	0.41 (0.09)^a^	0.58 (0.06)	0.37 (0.03)	0.43 (0.07)	0.50 (0.05)	0.32 (0.03)
	Exclude MHC SNPs	0.25 (0.09)	0.33 (0.06)	0.22 (0.03)	0.28 (0.08)	0.29 (0.05)	0.19 (0.03)
GCTA	All SNPs	0.39 (0.09)	0.42 (0.09)	0.26 (0.03)	0.27 (0.07)	0.34 (0.05)	0.20 (0.03)
	Exclude MHC SNPs	0.32 (0.09)	0.34 (0.06)	0.22 (0.03)	0.20 (0.07)	0.27 (0.05)	0.16 (0.03)

^a^The value in the brackets is the standard deviation of h^2^.

**Figure 2:**
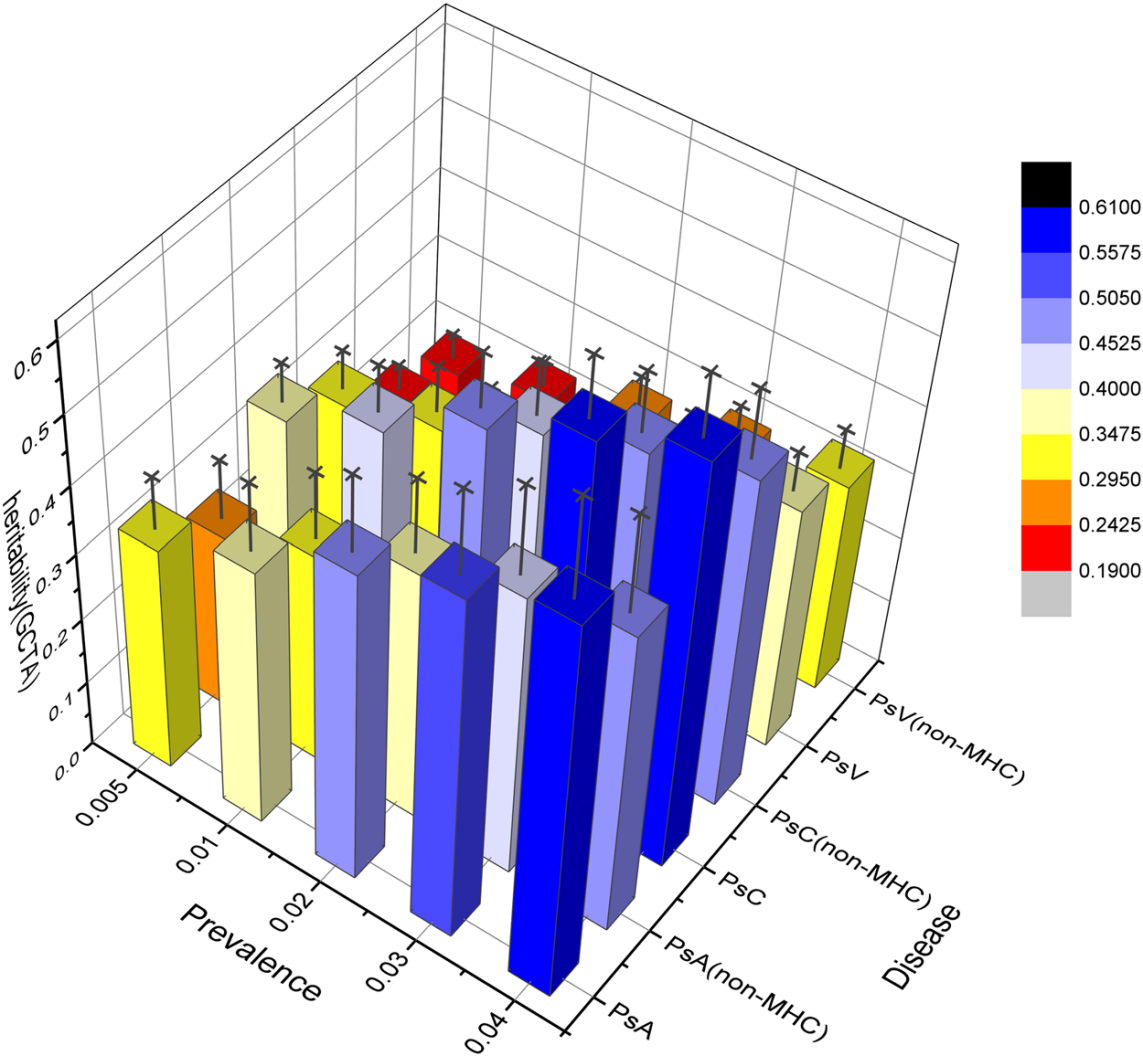
3D bar plot of heritability estimation including and excluding MHC SNPS using the GCTA method adjusted by prevalence. The SNPs are genotyped. The X-axis is disease prevalence from 0.5% to 4%, Y-axis is diseases and SNP regions, Z- axis is heritability score, standard deviation is showed as black error bar.

Using the GCTA method with 230k SNPs demonstrated an overall lower heritability of PsC (h^2^ = 0.42), PsA (h^2^ = 0.39) and PsV (h^2^ = 0.26) as compared with the LDAK method (Table 2). A similar trend was noted with SNP imputation (Table 2). Using the GCTA method with 401k imputed SNPs, the heritability of PsC (h^2^ = 0.34) was greater than both PsA (h^2^ = 0.27) and PsV (h^2^ = 0.20) (Table 2). The range of heritability estimates was smaller using the GCTA method as compared with LDAK (Table 2). The trend in hereditary (h^2^) estimates of the GCTA method were similar irrespective of disease prevalence or imputation (Table 2**;** Fig. 2). Similar to the LDAK method, a considerable proportion of the entire heritability was attributed to SNPs located within the MHC region (Fig. 2).

## Discussion

SNP-based GWAS studies offer an alternative approach to determining heritability of complex disease as it estimates the proportion of phenotypic variance explained by additive genetic factors. In this study, two such SNP-based methods were used to estimate the heritability using the genomic relationship matrix (GRM) in PsC, PsA and PsV.

Yang *et al*. used the restricted maximum likelihood algorithm (REML), implemented in the GCTA package, to estimate the proportion of variance in phenotypes from the genomic relationship matrix using the mixed-effects model^16,24,25^. This method was applied to common, binned SNPs to capture the amount of heritability caused by possible causal variants. In this model, the SNP effect should conform to a normal distribution without environmental interaction. Yang’s GREML approach is independent of LD; however, it may lead to bias when real causal variants have different distributions of MAF and LD compared with these associated or tagged SNPs^24^.

While the GCTA model assumes that heritability is independent of LD, Speed *et al*. hypothesized that heritability varies according to local levels of LD^17^. While the LD varies across the whole-genome, the LDAK model was designed to reduce redundant tagging and estimate the GRM by weighting the SNPs according to the individual LD. Unlike GREML, the LDAK model can correct some degree of overestimation.

Both methods have limitations for estimating the heritability of complex diseases^26^. The GREML methodology mainly captures phenotypic variation explained by SNPs that are correlated with genotyped SNPs in high LD with causative SNPs. Current GWAS genotyping arrays cannot sufficiently cover all genetic variants as rare causal variants with weak effects in low LD are poorly tagged by common SNPs. This can lower heritability estimation using GWAS data compared with pedigree or twin analysis. Second, the GREML methodology assumes the effect sizes are normally distributed. Bias will likely be introduced in the absence of normal distribution of effect sizes and gene/environment interaction. Other factors that could bias the estimation include the SNP MAF and LD. Recent studies^22,27,28^ have demonstrated that existing SNP-heritability estimate methods are sensitive to frequency- and LD-dependent factors. When using these approaches, it is prudent to proceed cautiously. Partitioning SNPs by MAF and LD can help provide a more accurate estimation of heritability^22^. Hou *et al*. recently estimated SNP-heritability without partitioning SNPs by MAF and/or LD^29^.

This study determined the SNP-based heritability of PsC, PsA and PsV through an unbiased estimate of phenotypic variance. Results from this study differ from population-based genetic epidemiological studies that report a much greater heritability for PsA than PsV^6,7^. SNP-based heritability estimates suggest a greater or equal heritability for PsC as compared with PsA. The higher heritability estimate for PsA compared with psoriasis (PsC, PsV) in previous epidemiological studies could be attributed to common environmental factors should be considered to account the strong recurrence rate of PsA over psoriasis among first degree relatives reported. The MHC effects on heritability is notable and stronger for PsC than PsA, leading to higher gene-based heritability estimates for PsC.

If we assume that the heritability estimates based on previous epidemiological studies are accurate, then what is surprising about GWAS findings to date in psoriatic disease is the absence of PsA-specific genes that have reached GWAS significance^3^. This is partly explained by the much larger number of patients in the PsC or PsV GWAS studies to date compared with PsA. Disease heterogeneity, which may be greater for PsA than PsC, particularly because the ascertainment criteria for PsC have been chronic plaque psoriasis, whereas PsA is more of a polyarticular, oligo-articular and axial disease. This may affect the heritability estimate for PsA. Finally, dominance and epistasis were not accounted for in our narrow-sense heritability (h^2^) estimate. The interaction between alleles at the various loci may have a significant effect on heritability estimates. Considerably increasing the number of PsA patients in GWAS studies will help clarify the heritability estimate question for PsA. A lower heritability for PsA or greater environmental influence on PsA should also be considered as possible explanations for these findings.

## Conclusion

Population- and clinic-based epidemiological studies have consistently documented significant familial aggregation in psoriasis and PsA. This is the first study to quantify the additive heritability of three subsets of psoriatic disease that is attributable to common susceptibility SNPs from large scale genotyping arrays. We noted that PsC, PsV and PsA were heritable. While the contribution of all SNPs resulted in greater heritability, a significant proportion of the heritability was attributed to SNPs located within the MHC region. Further, the heritability of psoriasis was notably greater than PsA in this study.
